# The work identity of leaders in the midst of the COVID-19 pandemic

**DOI:** 10.3389/fpsyg.2022.958679

**Published:** 2022-09-26

**Authors:** Stephanie Meadows, Roslyn De Braine

**Affiliations:** Department of Industrial Psychology and People Management, School of Management, College of Business and Economics, University of Johannesburg, Johannesburg, South Africa

**Keywords:** work identity, leader role identity, COVID-19 pandemic, social identity continuity, virtual leadership, role identity, leader identities

## Abstract

The world of work is being changed at an unprecedented rate as a result of the rise of the Fourth Industrial Revolution. This rate of change was accelerated by the COVID-19 pandemic, which left organizations and their leadership to deal with myriad of challenges. These changes also impacted leaders’ identities in their work and their roles in their organizations. We examine how leaders responded to the various workplace challenges presented by the COVID-19 pandemic and what this meant for their work identities as leaders. To do this, we made use of role identity theory, social identity theory, and leader identity. A qualitative study was conducted with a group of eight senior leaders from various South African and global organizations who had between five and 10 years’ work experience, and some had even more. Data were collected through semi-structured interviews, conducted virtually and in person. Thematic analysis was used to analyze the data. The main finding that emerged from the research was that leaders employed virtual leadership to ensure that customers’ expectations were met, and to manage team-and organizational performance. These leaders achieved this by fostering a digital culture and building effective teams. They achieved their leadership goals by ensuring social identity continuity amongst their teams. This required them taking on extra roles, such as strategist, technology expert, entrepreneur, coach, mentor, and member of the team. Their leader role identity, as part of their work identity, was amplified by the pandemic. The implication is that organizations should develop leadership development programs to increase and strengthen leader identities to capacitate them for times of crisis.

## Introduction

The COVID-19 pandemic ushered in unprecedented change for organizations globally. Leaders had to design fit-for-purpose operating models, which included new ways of doing things, new roles, and new ways of leading self and others (Jost et al., 2020).

COVID-19 was first identified in November 2019, in Wuhan, the capital of China’s Hubei province. The disease causes severe acute respiratory illness, which was sometimes fatal. The disease rapidly spread around the world, and no continent escaped the disastrous health consequences and socio-economic impacts.

Leaders worldwide were forced to design and implement strategies to deal with the pandemic at a very fast pace. National lockdowns were instituted globally, which compelled people to remain in their homes, in an attempt to control the spread of the virus. In South Africa, the economy slowed down, job losses increased, and other existing socio-economic challenges were exacerbated as a result of the lockdown regulations. During the lockdown period, the tools and technology of the Fourth Industrial Revolution (4IR) were implemented at an accelerated rate (High, 2020) to establish a “new way of working” (PricewaterhouseCoopers [PwC], 2020, p. 28). One of the immediate requirements during the COVID-19 pandemic was for organizations to ensure continuation of their business through virtual working, enabled by technology, and reskilling their employees. Remote workplaces became the “new normal,” which came with its own set of challenges for leadership and leadership identity.

Little is known about how a crisis like the COVID-19 pandemic affects the way leaders leads and the influence this has on their identities as leaders. There is a clear link between identity and leadership (Karp and Helgø, 2008), which is why, in this study, we examined how leaders responded to the various workplace challenges presented by the COVID-19 pandemic and, in particular, what this meant for their work identities as leaders. To do this, we made use of role identity theory, social identity theory, and leader identity theory.

### Work identity

Work identity can be considered a “multi-identity, multifaceted and multi-layered construction of the self” (Lloyd et al., 2011, p. 4). Gini (1998) posited that, fundamentally, “individuals form their identities through the work that that they do” (p. 707). Walsh and Gordon (2007, p. 5) defined work identity as a “work-based self-concept, comprised of a combination of organizational, occupational, and other identities, that affects the roles people adopt and the corresponding ways they behave when performing their work.” Popova-Nowak (2010), similarly, defined the concept as “a multidimensional work-based self-concept reflecting individual’s self-image that integrates organizational, occupational, and other identities shaping the roles and behaviors of individuals when they perform work” (p. 2).

Work identity is formed through the personal experiences, occupational skills, work context, work practices, and social memberships of an individual. It is considered “the importance of work for a person’s sense of self” (Bryan and Nandi, 2018, p. 1) and “how you define yourself through your engagement with various aspects of your work, such as your occupation, work-roles, and organization” (McQuaid, 2019, p. 1). Another definition is that a work identity is “a socially constructed representation of an individual’s unique self-perception of his/her own interactions within the employment environment” (Buche, 2008, p. 134). Caza et al., 2018) found that work identity causes individuals to perform their work in ways that reflect how they think and feel about their creativity, belief systems, and personal relationships with others.

### Social identity theory

Social identity theory describes how selves are personally, contextually, and socially derived (Sinclair, 2011). An individual’s self-concept is partly derived from the groups of which he or she is a part (Tajfel and Turner, 1985), which could include the departments and organizations in which they work and their professional affiliations. In this process, the self-concept becomes depersonalized (Brewer, 1991).

Organizations are social entities comprised of groups of people who collaborate and interact to perform work, and these groups of individuals develop and sustain socially derived identities (Sinclair, 2011). The identity of the group has a significant influence on the interests and motivations of group members (Sinclair, 2011). Membership of a group strengthens one’s position as a leader and a professional (Lloyd et al., 2011). Social identity allows individuals within groups to experience connection (Haslam et al., 2021). In the pandemic, it was shown that social identity continuity helped to reduce loneliness amongst employees (Krug et al., 2021).

### Role identity theory

Role identity theory seeks to explain the various roles we hold in our lives, and that “these roles come with prescriptions of how individuals should behave” (Van der Horst, 2018, p. 2). A role identity stems from an individual occupying or possessing a specific role (Farmer and Van Dyne, 2010). According to Walsh and Gordon (2007, p. 2), “a work identity is a reflection of the claimed central character of employees performing their work-related roles.”

If individuals have a strong role identity, they are more likely to take part in the role-based behaviors associated with the role, thus meeting role expectations (Farmer and Van Dyne, 2010). According to identity theory, role identities are formed as individuals work and interact with others in order to fulfill role expectations (Burke and Stets, 2009). One such role is that of leader.

How a leader’s identity is formed at work may also be explained through role theory (Kwok et al., 2018). Kwok et al. (2018) argue that leaders develop leader role identities when they perceive themselves as leaders, which influences the extent to which they behave in a leader-like way. This then strengthens others’ perception of them as leaders which leads to a stronger acceptance of them as their leaders.

### Leader identity and leadership identity

Leader identity is defined as “a sub-component of one’s identity that relates to being a leader or how one thinks of oneself as a leader” (Day and Harrison, 2007, p. 365). Leadership identities are defined as “experienced and projected selves or personas that aspire to “look” like leadership” (Sinclair, 2011, p. 509). Leaders are increasingly compelled to manufacture and project a sound and convincing sense of themselves, wanting to be seen as strategists and visionaries (Sveningsson and Larsson, 2014). Leaders are both “authors of and objects of identity production” (Sinclair, 2011, p. 509). This phenomenon is referred to as identity work (Sveningsson and Larsson, 2014).

## Materials and methods

A qualitative approach was used in this study, as we sought to understand the perceptions and related actions of leaders in the COVID-19 pandemic. Purposive convenience sampling, augmented by snowball sampling, was used, as it enables researchers to select participants who will make a valuable contribution to understanding the problem under study (Creswell, 2014, p. 239).

The data were collected using semi-structured interviews. This method was utilized because it offers flexibility by allowing new topics to emerge for exploration and further discussion (Saunders et al., 2009). Additional questions were posed to some participants to probe and obtain additional in-depth information. While some interviews were conducted in person, the majority were conducted virtually, using, amongst others, Microsoft Teams, Zoom, Google Meet, due to the COVID-19 pandemic restrictions. All interviews were recorded, with permission of the participants, using the cell phone application Voice Recorder Lite. The voice recordings were then transcribed using Otter.ai.

Participation was voluntary, and each participant signed an informed consent form. Anonymity and confidentiality were maintained throughout the research. Prior to the commencement of data collection, ethical clearance (#IPPM-2020-465M) was obtained the Ethics Committee of the Department Of Industrial Psychology and People Management at the University of Johannesburg.

The sample for this study consisted of a group of eight leaders from various South African and international organizations who led projects, business units, and/or teams during the COVID-19 pandemic. Table 1 provides the biographical information of the participants.

**TABLE 1:** Biographical information of participants.

No.	Gender	Job title	Years’ work experience	Industry
1	Female	Head of customer retention	20+	Insurance
2	Female	Head of department	40+	Private schools
3	Female	Head of distribution	20+	Fashion
4	Male	Head of data storage management	20+	ICT
5	Female	Head of commercial property: Africa regions	20+	Banking
6	Female	International affairs director	20+	Tobacco
7	Male	Managing director	20+	Management consulting
8	Female	Head of human capital management	30+	Consulting and public sector

The data were analyzed using thematic analysis, which is “a method for identifying, analyzing and reporting patterns (themes) within data,” and is widely used in qualitative research (Braun and Clarke, 2006, p. 79). Using an Excel spreadsheet, the steps proposed by Braun and Clarke (2006) were followed, which are as follows: familiarization with the data, whereby the transcribed data are read and re-read several times; noting key points; generating initial codes; and organizing the codes into meaningful groups, distinguished using color coding. The next phase was to search for themes, which involves taking a broader view of the data set (Braun and Clarke, 2006). Different sets of codes were combined to form a theme. Thereafter, the themes were refined or narrowed down by choosing the themes that appeared most in the data set and checking if the themes were aligned to the coded extracts and the entire data set. The themes were also refined by looking at the data in a particular code to see if there was a pattern that made sense (cf. Braun and Clarke, 2006). A further review was done on the entire data set, to see if the patterns that had emerged made sense. The last phase involved defining and naming the themes. Each of the themes was then checked and evaluated against the dataset and the research questions.

## Findings

Overall, participants related that the pandemic regulations introduced issues such as endless online meetings, burnout because of longer working hours, impacts on family life, negative effects on physical and mental health, increased customer needs, and supporting teams with issues such as fear of job loss, illness, and death in the family, working from home with limited office space, and stress. Many participants indicated that it was both challenging and enriching to lead themselves and their teams during this time. It is clear that the leaders occupied multiple roles during the COVID-19 pandemic in order to keep up with the change.

The detailed findings of this study are presented according to five themes, namely (a) Customer centricity, (b) Managing performance, (c) Building effective teams, (d) Building a digital culture, and (e) Work identity.

### Theme 1: Customer centricity

The *Customer centricity* theme emerged mainly when the participants were asked what they liked most about their role. The leaders in this study mentioned that it was key to understand customer needs so that they could build products and services that would satisfy their customers’ needs and expectations. The codes that emerged under the customer centricity theme were: (a) *understanding the business*, (b) *understanding customer needs*, (c) *building solutions for customers*, (d) *judgment and risk-taking*, and (e) *building great relationships with customers*.

For example, with regard to understanding the business, participants noted a renewed focus on certain aspects. Participant 1 said: “I like the outcomes of this role. Being able to retain the client, understanding the outcomes of the customer research, relooking product information and pricing in order to retain clients, and achieving customer retention targets for a large insurance company is satisfying.” Statements to support this included “I have to meet my customers delivery dates” (Participant 3) and “We used basic technology to help with research to understand why our customers were leaving us” (Participant 1).

Many participants indicated the need for building solutions for customers, for example: “In an ever-changing environment, the organization looks to its leaders to provide the solutions during times of change. I must provide strategies and solutions” (Participant 4).

With regard to judgment and risk-taking, some leaders indicated that, in a highly regulated environment such as banking or insurance, there is little room for judgment and decision-making, and that this can be frustrating when it slows down delivery and meeting customer expectations. Participants indicated that they preferred a work environment where they are empowered to use their judgment in making decisions in a collaborative way, provided they do not place the business at risk. In support of this, Participant 1 stated: “I would make a judgment call and go outside the boundaries, as long as it was legal and not putting the business at risk …. There has to be room for strategic thinking, planning, and flexibility to allow for non-conventional decision-making, and I must be able to use judgment in decision making.” Participant 8 stated: “I like an environment where people are empowered and expectations are clear.”

Concerning building great relationships with customers, participants indicated that key to business success is the ability to remotely build relationships with customers and partners. Participant 6 proudly noted: “I am a trusted business partner,” while Participant 5 said: “I build great relationships with my client base.” However, some participants indicated that building customer trust was very challenging during lockdown.

### Theme 2: Managing performance

Leaders in this study mentioned they had to assume the role of performance manager for their organization, their team, and for themselves. In addition to this, they had to also see to the wellbeing of their individual team members. According to Participant 8, “COVID has affected people’s mental health. It has affected their ability to engage externally, it has affected their ability to even engage in the company. So, now you must pay much more attention to mental health and wellbeing instead of staff performance only.” Participants stated that, during the COVID-19 lockdown, they were “dealing with staff wellbeing…, especially mental wellbeing” (Participant 5), and “loss of loved ones, general fear, and manual processes” (Participant 8). This led to participants adjusting the way in which they managed staff performance and led during this period. This theme is supported by the codes (a) *organizational strategy*, (b) *setting clear goals and expectations*, (c) *providing open and honest feedback*, (d) *coaching*, (e) *acknowledging one’s team*, (f) *building relationships with the team*, and (g) *trust.*

With regard to organizational strategy, participants indicated that they had to get involved in formulating strategy in a collaborative way with internal and external partners. This is evidenced by the following extracts: “I spend an awfully long time coaching rather than leading. I play different roles—strategy, coaching, implementation, alignment. My brain had to change six or seven times a day, depending on what kind of call I have” (Participant 6).

Leaders indicated that setting clear goals and expectations was key in managing their own performance and that of their team, and that this aspect became crucial during lockdown, when teams had to work virtually. Participant 2 noted having to get “something sorted out” to meet targets. Participant 1 mentioned a focus on “outcomes of the role, being able to retain the client, achieving the targets.”

The participants indicated that, in building an effective team, providing open and honest feedback to their peers, their managers, and the people who report to them is crucial in achieving desired outcomes that would ultimately address customer needs and ensure achievement of performance objectives. This was made clear through statements such as “I must have a positive attitude, let go and move on, be open and honest, have no hidden agenda, and provide honest feedback” (Participant 3), “I will confront issues that I believe needs to be changed for the good. I will speak my mind” (Participant 2), and “Coaching becomes very key in giving real-time feedback” (Participant 7).

The code coaching was mentioned several times by participants during the discussion of team performance. This code was mentioned by two participants as the element they enjoyed the most in their job. Participant 7 had taken on “coaching people to be their best in their current role,” while Participant 6 stated, “I spend an awfully long time coaching rather than leading.” Participant 2 said that she had enjoyed the “opportunity to display natural leadership, interacting with people, giving guidance to others, setting the example, coaching, and mentoring.” Participant 8 indicated: “I have check-ins with the team once a week, sometimes impromptu meetings, not about work, but about their lives. I create a platform for people to discuss their issues—mental health, dealing with loss of family due to COVID-19, fear of losing jobs, combining home life and work life while working from home, etcetera” (Participant 8).

The participants all mentioned the importance of acknowledging one’s teams, and most of the participants spoke at length about the importance of having a high-performing team and the leader’s role in building such teams. Participants indicated that their ideal work environment was one where everyone understood what they must do, and that they do it. Participant 4 stated, “What I like most in my role is team involvement and teamwork,” and added: “I view myself as one of the guys, as a team member. I don’t enforce my position.” Participant 2 stated: “What I like most in my role is the opportunity to display natural leadership, i.e., being a leader without necessarily having a leadership title, interacting with people, giving guidance to my team, setting the example, coaching, and mentoring.”

When participants were asked what they enjoyed least about their role, the trust code emerged. Leaders all indicated in some way that building trust was vital. Participant 3 indicated that she trusted her team, but that this became challenging when employees worked virtually. This meant working harder to build trust: “I do trust them, but I know the staff well-enough to know that they’re not going to do the work when you need them to do it. So, I’ve got to double check, unfortunately.” Participant 6 noted: “I think the biggest challenge is where you have relationships, where you have established, mature trusted relationships before COVID. Those relationships continue, and you can continue to build on them virtually. But, where you do not have relationships, it’s almost impossible to build new ones, and that is the impact of COVID.”

### Theme 3: Building effective teams

Building effective teams is a theme supported the following codes: (a) *effective leadership*, (b) *changed leadership style*, (c) *resilience*, (d) *making decisions*, and (e) *upskilling teams*.

With regard to effective leadership, Participant 3 described the challenges of managing a team in this time, stating: “There are many moving pieces in the shipping logistics world. This aspect became even more challenging during COVID-19. It is very stressful.” Participants indicated that certain leadership attributes have become more important than ever. “I lead from the front, and I am an entrepreneurial leader. I have received award for being an innovative leader and doing things differently during COVID,” said Participant 5. Participant 8 stated that she was “an inspiration to my team. I expect a lot, I am very self-aware,” while Participant 1 described herself as “committed, dedicated and passionate.” Participant 2 stated, “I want to influence horizontally, I am assertive, I express myself.” Participant 3 mentioned, “I lead by example; I make sure I am available for my team, and I jump in and work with them.” Participant 5 indicated: “I led from the front during this crisis, I met all my people online, managed the team electronically, got in the same room at the same time, built new portals… Find new ways of doing things—new financial checks, deal with uncertainty, deal with sickness, losing loved ones etcetera, this is leadership on another level.”

The code changed leadership style emerged from participants’ discussions about how they dealt with the new challenges. They indicated that they had had to change the way they led, and that they had to learn new ways of doing things. For example, Participant 7 commented: “Managers are having to deal with the wellness component. “Is the person that I’m dealing with, okay?” “Are they feeling alone?” “Are they heading for burnout?” “Are they stressed?” As a leader, that’s another element that you have to manage” (Participant 7). A number of participants supported the sentiment of Participant 3, who noted: “Before COVID, I was not a hovering manager, because I was with the team. Now, I have to check in all the time to see if work has been done. I don’t like doing it, but I have to make sure it is being done.” Participant 6 mentioned the positive aspect of now “having a better understanding what the impact of COVID is on your business. We know for example, how many people [staff members] are sick—we get a weekly update. Participant 5 advised: “Lead with humanity.” Participant 3 noted that “being able to manage people remotely requires a mindset change to manage people where you don’t see them.” Other participants expressed similar changes in mindset: “I have to define boundaries; for example, I said to my team, “No meetings, after a certain time.” I am flexible with my team; it’s a give-and-take mindset. Different people’s work styles require different approaches. Some people need to be micro-managed: “Now you have to sit down” … schedule teams meetings, talk to them about the fact that they have to be self-reliant. We don’t have time to micro-manage people. Also, you’re getting involved in their personal lives … must approach people more holistically… more empathetic. More understanding is required, not only a manager of work, but of context and socio-economic conditions” (Participant 8).

Participants noted that resilience, both their own and their team’s, was vital in coping with the new uncertainties and fears. Participant 5 noted: “It has taught me that human beings are wired up to just cope regardless of the odds. The best leaders led their teams to find ways to cope during this time.” Participant 5 noted the importance of developing resistance, both your own and that of others.

With regard to making decisions, most participants indicated that, to effectively lead self, others, and the organization, they had to make decisions quickly. However, some participants indicated their frustration with being in a highly regulated environment (such as banking and insurance), where quick decision-making is vital in order to attract and retain customers.

Many participants noted the importance of upskilling teams to effectively deal with the challenges brought about by the pandemic. Participant 2 explained: “We experienced challenges mostly in the beginning of COVID-19, where staff did not have access to online tools. We had to ensure tools are available and staff are proficient in using the tools.” Participant 5 stated: “I think leaders must be honest about their lack of skills and ask for help,” while Participant 7’s strategy was to “learn on the go.”

### Theme 4: Building a digital culture

The crisis brought about by the pandemic forced these leaders to fulfill their roles through a digital culture. The theme is supported by the following codes: (a) *emerging technology*, (b) *new ways of working*, (c) *work–life balance*, (d) *ideal work environment*, and (e) *a new way of leading*.

With regard to emerging technology, some of the leaders indicated that only through emerging technology could they perform their work during the lockdown. They indicated that it was a steep learning curve, but one that was well-worth it. “Technology is the only reason I can work,” admitted Participant 6. She continued, “I have a laptop, I have unlimited data, and the company is migrating us from our current phones to iPhone 12 … She noted that she was able to migrate to the new phone “with zero support from IT,” adding, “Everything that is transactional can now be done in a digital way.” Participant 1 also noted the ease of technology: “Technology has made my work easier; many processes were smoother and faster. It allowed me to learn new skills and multi-task.” Participant 5 noted the need for technology “to make people-related decisions to enhance organizational performance, and added: “I have had to reflect on my ability to utilize technology. I have had to upskill myself.”

New ways of working came up frequently, with leaders noting that emerging technology and COVID-19 ushered in new ways of working, where virtual working in a flexible way became the norm, requiring continuous learning, adaptability, collaboration, and a change in mindset. Participant 5 remarked: “I’m not a big techie…, but I have to learn to do things differently. Everything is fully automated and, in property, one now needs to understand how to fund green buildings. Drone technology is also used to see and inspect our properties … but, at the end of the day, one must walk the assets to get a real feel for the property. It has been a massive disruptor, and I am still figuring out if I really like it or not (Participant 5). Participant 2 also noted the impact of technology: “I had to learn new ways of doing things very fast, for example, Google Classroom. This also meant dealing differently with class discipline. I had to provide a blended approach to learning.” Participant 1 noted: “It allowed me to learn new skills better and multi-task. It also made my work easier. Many processes were smoother and faster.”

Most leaders found that technology and working virtually positively impacted their work–life balance. “There is now technology to enhance what I am doing, and it afforded flexible working hours” (Participant 1). Participant 4 noted: “It has had an impact on work–life balance, since I spend more time with my family.”

Leaders shared their views of an ideal work environment, with Participant 8 commenting: “I like an environment where people are empowered and expectation is clear” (Participant 8). “Red tape was diminished; processes were optimized” (Participant 2). Participant 6 suggested “30% in the office, 30% with external stakeholders, 30% in the field with your people, and then 10% thinking, reflecting, reading.” Participant 6 also expressed concern around the over-digitizing of the work environment, which leads to culture of anonymity.

### Theme 5: Work identity

Participants had to adopt several roles during the COVID-19 pandemic, which affected their work identity. The theme is supported by the following codes: (a) *strategist/visionary*, (b) *entrepreneur*, (c) *high performer*, (d) *one of the team*, (e) *influential*, and (f) *self-aware*.

The participants found that one of the new job roles that they were expected to participate in was that of strategist/visionary. Participant 6 mentioned: “I play different roles—strategy, coaching, implementation, alignment. My brain changes six or seven times a day, depending on what kind of call I have. Participant 4 also indicated that he has to get involved in developing the vision and strategy to produce new ideas for his organization, in collaboration with others: “In an ever-changing environment, the organization looks to its leaders to provide the solutions during times of change. I must provide strategies and solutions. What makes it easier is that we collaborate with our partners to assist with this task.”

With regard to being an entrepreneur, the participants, particularly Participant 5, noted the importance of an entrepreneurial leader: “I innovate and have innovated during 4IR and COVID-19 pandemic—doing things differently.”

Participants encouraged their teams to be high performers by role-modeling the behavior. As Participant 5 put it: “I lead from the front.” Participant 3 noted: “I lead by example. I make sure I am available for my team, and I jump in and work with them.”

Participants indicated that they became one of the team to achieve objectives. Participant 4 explained: “I view myself as one of the guys, as a team member. I don’t enforce my position. I speak Afrikaans and English to accommodate the team members.”

A number of participants indicated that an aspect of their work identity was being an influencer. “I influence horizontally, I am assertive, I express myself” (Participant 2). Participant 8 noted: “I am an inspiration to my team. I expect a lot. I am very self-aware.”

## Discussion

The present study explored how leaders responded to the workplace challenges presented by the COVID-19 pandemic and what this meant for their work identities as leaders. To do this, we made use of role identity theory, social identity theory, and leader identity theory. The leaders in this study had mainly two broad responsibilities: first, to ensure that they performed in their roles as they did before the pandemic and delivered on objectives, and, second, maintained team cohesion and performance. This they had to achieve in the context of remote working and virtual leadership.

Recent research indicates that many companies made use of virtual leadership to maintain their organizations during this period (Bizilj et al., 2021). Leaders had to learn new ways of doing things, and also had to guide their teams in learning new ways of doing things, all while maintaining a fast pace. This supports the findings of Högberg (2022). Participant 5 summed it up as follows: “We adjusted.” The pandemic forced the leaders to upskill themselves, which further enhanced their work identities as leaders, as learning opportunities are known to strengthen work identities (Collin, 2009; De Braine and Roodt, 2011). Leading from a distance presented additional challenges to the leaders, in that they had to secure the trust in existing relationships and build trust relationships with new employees, customers, and peers.

As part of overcoming challenges, the leaders fostered a digital culture to ensure that customer expectations were met and team performance was managed. Most welcomed technology and used it to enhance delivery of products and services. One participant referred to the threat of a culture in which people become “anonymous” as a result of over-digitizing procedures, and noted that this was not ideal when attempting to build effective teams. Some leaders focused on building and enhancing teamwork to compensate for the challenges that came with over-digitizing procedures.

Technology changes the organizational context, which reshapes work identities. Technology artifacts become standards in “self-narratives” when the function thereof aligns with the work identity of an individual (Stein et al., 2013, p. 178). Individual, in this case, the leaders, position themselves and others in relation to the technology artifacts, and a preferred self is expressed (Stein et al., 2013). The roles and behaviors of these individuals were altered as their use of information communication technology increased, and their work identities were reshaped on a continual basis. This reshaping of the identity through the use of technology is also shaped through technological interactions with others (Piszczek et al., 2016). The role of leadership is thus to support and align with technology to reduce potential threats to employees’ identities (Mirbabaie et al., 2022). This would then also influence team performance.

One of the main themes was building effective teams, which, according to one participant, entailed becoming “one of the team.” This required self-awareness. By becoming one of the team, the way a leader strengthens his/her position as a leader and as a professional is now informed by a different or additional team members. By leading the team and being one of the team, it is argued that the leaders helped to strengthen the social identity continuity for themselves and their employees. In a study on leaders and their employees during the COVID-19 pandemic, social identity continuity was found to be related to job satisfaction (Krug et al., 2021). As work identity is a form of social identity, we argue that, as the leaders become “one with their teams,” their work identities are enhanced and strengthened. This was achieved through the leaders employing identity entrepreneurship, which entails the behaviors that bring about group cohesion (Reicher et al., 2005). Ultimately, a new role is added to their work identity.

Work identity is multidimensional, consisting of roles (Walsh and Gordon, 2007) or “selves,” which are now being added at a much faster pace than before. Leader identity is also regarded as social identity (Hogg, 2001). The leaders under study exerted their leadership through the roles and relationships they had with others. Adopting new roles and new ways of doing things greatly influenced their work identities. In addition, they also viewed themselves as strategists, technology experts, entrepreneurs, coaches, mentors, and a member of their team.

The participants shared that their leader role identity, as one of the multiple facets of their work identity, emerged stronger during the COVID-19 pandemic. Their aim was to ensure effective staff performance and, ultimately, organizational survival. This is aligned to the view of Kwok et al. (2018) that leader identity impacts outcomes such as organizational performance and customer retention.

The leaders under study were also required to deal with matters related to employee wellness and work–life balance. As a result, many of the leaders took on the extra role of coach to guide their teams through personal struggles such as the loss of family members, the fear of losing their jobs, illness, and longer working hours. The pandemic had a tremendously negative effect on the wellbeing of employees (Krug et al., 2021; Mähring et al., 2021), and the participants indicated that building an effective team required that they behave differently as leaders.

### Theoretical contribution of the study

The main finding that emerged from the research is that the leaders fostered virtual leadership to ensure that customer expectations are met, and to manage and enhance team-and organizational performance. They achieved their leadership goals by ensuring social identity continuity amongst their teams. To do this, they had to take on extra roles, such as strategist, technology expert, entrepreneur, coach, mentor, and member of their team. Based on these findings, we argue that leader identity, as part of a leaders work identity, is enhanced and lived out by leaders ensuring social identity continuity amongst their teams and fulfilling the respective leadership roles that come with their leader role identity. This study has linked role identity, leader identity, and social identity theory with work identity in examining the ways in which leaders lead and identify with their leadership role during a time of crisis.

#### Methodological implications

There are two methodological implications in this study, which could also be considered limitations of the study. The first one, being that most of the semi-structured interviews were conducted using online platforms, due to the COVID-19 pandemic restrictions. As a result of this, important social cues that are easier to pick up on in face-to-face interviews may have been missed. Secondly, six of the participants were female and only two were male, as a result of purposive convenience and snowball sampling may have created gender-bias implications.

### Policy and practical implications for organizations and HR

Organizations should focus on the development of leaders’ work identities in their leadership development programs and initiatives, coupled with taking into consideration the multiple complexities associated with leadership roles. Aspects such as role congruity need to receive greater focus for development programs, to create better leader–job fit, which ultimately would enhance leader-role identity and overall work identity. There should also be a focus on crisis management within the broader organizational context. The study revealed the importance of leadership playing a role in ensuring the wellbeing of their employees, and HR practitioners and leaders therefore need to prioritize organizational initiatives around wellbeing during times of crisis.

#### Limitations of the study

As this was a qualitative study, the findings cannot be generalized to the greater population. This study consisted of a sample of eight leaders from different organizations.

### Recommendations for future studies

Future studies should be conducted on a larger sample with participants from multiple countries, in order to gain a broader understanding of identity formation of virtual leaders in times of crisis. Researchers should ensure a representative sample, which would yield insights according to gender and ethnicity. Future research could also be longitudinal, in order to gain an understanding of developments over time. Such research could include quantitative instruments to complement the data.

## Conclusion

The leaders who participated in this study had a stressful task in leading and creating social identity continuity amongst their teams in order to lessen the effects of the COVID-19 pandemic. This was achieved by virtual leadership *via* technology. In this process, leader role identity, as one dimension of the work identity, emerged more strongly, with the leaders viewing themselves as strategists, coaches, and team members. They had to learn to lead differently. As the new world of work continues, leaders need to strengthen their role identities as leaders, in other words, their leader self-concept, to lead effectively in times of change and crisis. In this way, leaders will be able to strengthen the social identity of their teams and followers.

## Data availability statement

The datasets presented in this article are not readily available because the data has not been made publicly available due to the restrictions imposed as a result of privacy and ethics. Requests to access the datasets should be directed to RD, roslynd@uj.ac.zal.

## Ethics statement

The studies involving human participants were reviewed and approved by Ethics Committee of the Department of Industrial Psychology and People Management, School of Management, College of Business and Economics, University of Johannesburg, ethical clearance number: IPPM-2020-465(M). The patients/participants provided their written informed consent to participate in this study.

## Author contributions

This article was adapted from the Master’s mini-dissertation of SM, who conducted the research. RD was the study leader, and provided conceptualization guidelines and editorial inputs. Both authors contributed to the article and approved the submitted version.
